# Computational properties of mitochondria in T cell activation and fate

**DOI:** 10.1098/rsob.160192

**Published:** 2016-11-16

**Authors:** Roman Uzhachenko, Anil Shanker, Geneviève Dupont

**Affiliations:** 1 Department of Biochemistry and Cancer Biology, School of Medicine, Meharry Medical College, Nashville, TN, USA; 2 Host–Tumor Interactions Research Program, Vanderbilt-Ingram Cancer Center, and the Center for Immunobiology, Vanderbilt University, Nashville, TN, USA; 3 Unité de Chronobiologie Théorique, Université Libre de Bruxelles, CP231, Boulevard du Triomphe, 1050 Brussels, Belgium

**Keywords:** mitochondria, Ca^2+^ signalling, PID controller, digital and analogue signals, T lymphocytes, systems biology

## Abstract

In this article, we review how mitochondrial Ca^2+^ transport (mitochondrial Ca^2+^ uptake and Na^+^/Ca^2+^ exchange) is involved in T cell biology, including activation and differentiation through shaping cellular Ca^2+^ signals. Based on recent observations, we propose that the Ca^2+^ crosstalk between mitochondria, endoplasmic reticulum and cytoplasm may form a proportional–integral–derivative (PID) controller. This PID mechanism (which is well known in engineering) could be responsible for computing cellular decisions. In addition, we point out the importance of analogue and digital signal processing in T cell life and implication of mitochondrial Ca^2+^ transport in this process.

## 1. Introduction

Activation of T lymphocytes is a complex process associated with remodelling of signalling network and metabolism. Initiated by the triggering of T cell receptor (TCR) at the plasma membrane, activation propagates into the nucleus, resulting in transcriptional changes. Obviously, the amplitude and duration of T cell activation should be tightly controlled owing to undesirable outcomes of under- or overactivation, such as immunodeficiency or autoimmunity, respectively. Furthermore, T lymphocytes should respond adequately to the type of pathogen (i.e. viral, bacterial, tumour) and to its dose. Finally, T cell memory serves to fight the repeated pathogen encounters and needs remodelling of metabolic processes to meet re-activation requirements.

Studies of the last 10–15 years shed light on metabolic reprogramming in T cells. Currently, it is well accepted that resting T cells mostly use oxidative phosphorylation and beta-oxidation to retrieve energy from nutrients. However, upon activation, T cells switch from respiration to more active glucose and glutamine uptake, as well as aerobic glycolysis [1,2]. The latter serves for active production of monomers required for nucleic acid synthesis and is in turn necessary for cell proliferation and expansion of specific T cell clones [3,4]. This effect is reminiscent of the well-known Warburg effect [2].

Recent data in mitochondrial biology allow us to consider mitochondria simply as cellular energetic stations, but also as important signalling hubs. Indeed, first results in this field came from findings that mitochondria produce reactive oxygen species (ROS) and communicate with the nucleus via oxidative modification of proteins including transcription factors [5–8]. Another important step forward was made after the identification of the molecular nature of the mitochondrial Ca^2+^ uptake/uniport (mtCU). Although the existence of a process of Ca^2+^ inward transport (from cytosol into mitochondrial matrix) was known for at least 50 years, the pore-forming and the Ca^2+^ sensor regulatory subunits of the mtCU, mitochondrial Ca^2+^ uniporter (MCUa) and mitochondrial Ca^2+^ uptake 1 (MICU1), respectively, have been described for the first time only a few years ago [9–12]. Currently, the role of several more proteins (such as MICU2–3, MCUb, EMRE, MCUR1, UCP2) involved in the control of mtCU has been described [13–17]. Finally, NCLX1 (Na^+^/Ca^2+^ exchanger 1) was identified as a protein molecule responsible for Na^+^/Ca^2+^ exchange, a process that balances mtCU-induced Ca^2+^ accumulation and prevents matrix Ca^2+^ overload, which could potentially lead to apoptosis [18,19]. Although the MCU and NCLX1 are the prominent and best identified pathways for Ca^2+^ influx and efflux in and out of mitochondria, other pathways have been reported to play a role in these exchange processes [20].

**Figure 1.**
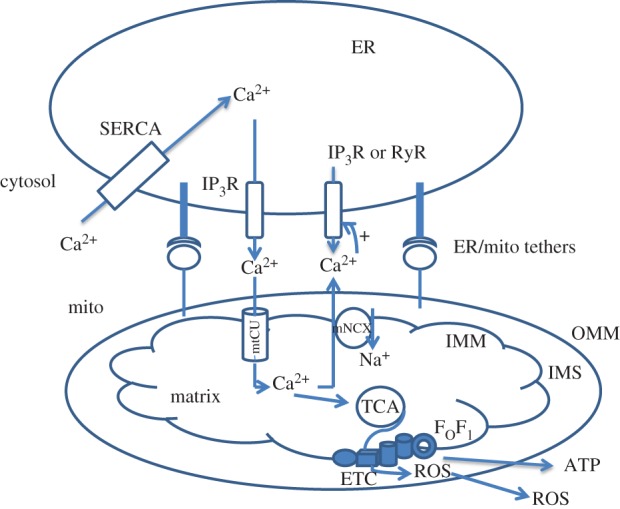
General scheme of Ca^2+^ exchange between the endoplasmic reticulum and mitochondria. Multiple proteins form tethers controlling the ER/mitochondria junction and maintaining ER/mitochondria Ca^2+^ crosstalk. Ca^2+^ release induced by the opening of RyRs or IP3Rs leads to elevation of Ca^2+^ at the ER/mitochondria interface. When Ca^2+^ reaches a concentration of 3–10 µM (threshold for mtCU), the MCU opens and allows Ca^2+^ ions to be imported into the matrix where they stimulate the tricarboxylic cycle (TCA). As a consequence, the electron transport chain (ETC) starts producing ROS, which are utilized by antioxidant systems. At the same time, Ca^2+^ ions are directed from mitochondria into the cytosol by the mitochondrial Na^+^/Ca^2+^ exchanger (mNCX) at the expense of Na^+^ import. This Ca^2+^ activates IP3Rs and RyRs in the ER/mitochondria space, thereby leading to Ca^2+^ release from the ER. OMM, outer mitochondrial membrane; IMM, inner mitochondrial membrane; IMS, intermembrane space.

Further studies in mitochondrial biology progressed towards the understanding of interorganelle interactions, in particular Ca^2+^ transfer between the endoplasmic reticulum (ER) and mitochondria that is mediated by molecular bridges (figure 1). Major players that form ER/mitochondria tethers are Grp75/HSPA9, inositol trisphosphate receptor (IP3R), mitofusin 2 (MFN2) from the outside surface of the ER and the voltage-dependent anion channel 1 (VDAC1), and mitofusins MFN1 or MFN2 from outside mitochondrial membrane (OMM) [21]. The presence of physical contacts between the ER and mitochondria explains the paradox of mitochondrial Ca^2+^ accumulation: mtCU has an intrinsic low affinity (i.e. mitochondria import Ca^2+^ only after significant Ca^2+^ elevations around 3–10 µM), whereas most of global cellular Ca^2+^ spikes do not reach this level [15,22–24]. This apparent discrepancy was resolved by showing that protein complexes tether ER to mitochondria, thus close positioning these organelles and creating local Ca^2+^ domains with ion concentration of 10 µm and above [25–27]. Thus, the ER–mitochondria interface represents a local signalling platform where cytosolic Ca^2+^ elevations are transmitted to mitochondria [28,29] (figure 1).

Mitochondria are able to accumulate large amounts of Ca^2+^ ions, but the increase in free Ca^2+^ induced by cytosolic Ca^2+^ transients is limited by buffering by anions present in mitochondrial matrix, such as phosphate (Pi), acetate, bicarbonate, etc. [30,31]. Mitochondrial Pi buffers sink extra amounts of Ca^2+^ until reaching the buffer capacity; after this point, Ca^2+^ phosphates trigger mPTP opening [31,32]. mtCU and Pi buffer work cooperatively to respond to cytosolic Ca^2+^ increases. If rapid and submicromolar concentrations of cytosolic Ca^2+^ elevations induce fast accumulation of free matrix Ca^2+^ (mCU1 mode), which upregulates electron-transport chain (ETC; see below), then sustained mtCU activation (mCU2) leads to the expansion of the compartment of matrix Ca^2+^ sequestered by phosphate buffer [31].

How might all these molecular mechanisms be related to T cell functions, activation and fate? The most obvious hypothesis is that mitochondria participate in the supply of ATP in activated T cells, and in turn in ATP-dependent transport processes in immune synapse, where the majority of signalling events are triggered [33,34]. Second, mitochondria may be involved through the opening of the mPTP in its high-conductance mode. Mitochondria are indeed involved in apoptosis induction during activation-induced cell death caused by TCR stimulation and aimed at restricting further clonal expansion to prevent autoimmune disorders [35,36]. Finally, mitochondria produce ROS, which accompany upregulation of nuclear factor kappa B (NFκB) and nuclear factor of activated T cells (NFAT), two master regulators of TCR-stimulated transcription changes [37–41]. These three processes are stimulated by an increase in mitochondrial Ca^2+^, as this ion stimulates the tricarbon acid (TCA) cycle in the mitochondrial matrix, leading to an increase in proton pumping, ATP synthesis and ROS production [42,43] (figure 1). It is noteworthy that the mtCU inhibitor, Ru360, prevents ROS formation in activated T lymphocytes, thus showing that the mtCU is the mechanistic link between TCR-induced Ca^2+^ elevation and ROS production for mitochondria–nucleus communication [40]. In this review, we propose that mitochondrial Ca^2+^ transport and mitochondria could also play a key role in the central regulation of T cell fate. In addition, we try to understand why activated T lymphocytes use mitochondria for cell decisions, in particular from the perspective of Ca^2+^ transport.

## 2. The importance of mitochondrial Ca^2+^ for T cell life and fate

The importance of the mtCU in physiology and pathology of T cells is demonstrated by several different examples. Components of Ca^2+^ signalling machinery in T cells, ryanodine receptors (RyR), IP3R and Orai1 channel subunits mediating store-operated calcium entry (SOCE), are coordinately associated with MCU expression; downregulation of these proteins in activated T cells leads to a compromised upregulation of the MCU mediated by the CREB transcriptional factor [44]. The significance of mitochondria in the control of cytosolic Ca^2+^ turnover has been shown for the Jurkat T cell line: dissipation of mitochondrial membrane potential (ΔΨm), the driving force for mtCU, causes early Ca^2+^ release-activated channel (CRAC) inactivation (discussed below), Ca^2+^ signal reduction and decreased nuclear import of NFAT [45]. Apparently, changes in SOCE activation via elevated mtCU mechanism may explain the fact that T lymphocytes from systemic lupus erythematosus display sustained increased Ca^2+^ response after their activation, which is proposed to be triggered by upregulated nitric oxide-dependent mitochondrial biogenesis [46,47]. Protein p13 of human T cell leukaemia virus type 1 (HTLV-1) modulates ΔΨm, thus limiting Ca^2+^ uptake and affecting T cell activation and death [48]. Further, we showed that in CD4^+^ T cells lacking mitochondrial tumour suppressor Fus1/Tusc2 with a potential Ca^2+^-binding domain (also discussed below), upregulation of NFAT- and NFκB-driven genes after activation was diminished [49]. An interesting role of the mtCU-mediated ROS production in negative feedback regulatory loop has been described in T cells: mitoROS produced by elevations in cytosolic Ca^2+^ stimulate protein kinase D, which in turn activates DRAK2, thus negatively regulating the influx of external Ca^2+^ [50]. Considering the ER/mitochondria Ca^2+^ crosstalk, new linkers with profound effect on T cell responses have been identified. In T cells, transglutaminase 2 (TG2) binds RAP1, the guanine nucleotides exchange factor of small GTPases, which enhances ER Ca^2+^ release and induces mitochondrial Ca^2+^ accumulation [51]. The absence of TG2 led to a diminished mouse CD8^+^ T cell activation and memory cell formation *in vivo* [52]; in Jurkat cells, TG2 overexpression leads to apoptosis *in vitro* [51]. Another protein, Tespa1, is localized closed to ER/mitochondria contacts, and directly interacts with Grp75 and IP3R [53]. Mitochondrial GTPase of the immune-associated nucleotide-binding protein 5 (GIMAP5) links mitochondria with microtubule cytoskeleton, helping them to localize at sites with high Ca^2+^ concentration and to maintain membrane Ca^2+^ currents via SOCE, thus favouring T cell survival [54]. Finally, mitochondrial Ca^2+^ accumulation triggers ATP synthesis and secretion via *panx1* transporter in the activated Jurkat and human CD4^+^ T cells, whereas secreted ATP activates P2 purinergic receptors involved in the maintenance of intracellular Ca^2+^ elevation [55]. These examples are convincing about the diversity of roles of mitochondrial Ca^2+^ in T cell life and fate.

However, the significance of mitochondria in T cell activation and homeostasis will not be fully appreciated without quoting mitochondrial dynamics, a process of mitochondrial remodelling via fusion, fission, autophagy and movement [56]. These processes are tightly controlled by GTPases: mitofusins 1 and 2 and OPA1 for fusion, DRP1 and its receptor Fis1 for fission, and finally Miro 1 and 2 for movement [57–59]. Ca^2+^/calmodulin (CaM) kinase I alpha phosphorylates DRP1, leading to its binding with Fis1 and mitochondrial fission [60]. Other Ca^2+^-dependent proteins, Miro 1 and 2 possessing Ca^2+^-binding domains called EF-hands, promote mitochondrial fragmentation after interaction with Ca^2+^ ions [61]. In T cells, TCR triggering is associated with translocation of mitochondria to the immune synapse controlled by DRP1, which positions fragmented mitochondria in close proximity to the peripheral supramolecular activation cluster (pSMAC) [62]. Upon stimulation and supplying the immune synapse with ATP, CD3 molecules providing TCR proximal signalling move from pSMAC towards central SMAC and become internalized. In the absence of DRP1, CD3 molecules remain in the immune synapse and continue sending signals inside cells, thus increasing TCR response strength (e.g. IL-2 synthesis) [33]. It is noteworthy that intensity of TCR-triggered signalling defines Th polarization with mostly Th1 differentiation upon strong stimulation [63]. Obviously, the ability of mitochondria to take up Ca^2+^ should affect the processes of mitochondrial dynamics similar to other Ca^2+^-dependent processes such as activation of PKB/Akt [64] and NADPH oxidase [65].

Interesting connections between mitochondrial dynamics, respiration and metabolic reprograming have been recently described for T cells facing the choice between effector (Te) and memory (Tm) phenotypes. In particular, it was demonstrated that downregulation of DRP1 function leads to mitochondrial fusion-favouring activation of respiration over aerobic glycolysis in Tm cells, whereas the opposite effect was observed in Te lymphocytes [66]. It is noteworthy that DRP1 is activated by phosphorylation while its inhibition is associated with dephosphorylation by Ca^2+^-dependent phosphatase calcineurin (CaN) [67]. In this respect, modulation of Ca^2+^ signals including mtCU would coordinate mitochondrial dynamics with metabolic demands and execute metabolic reprogramming.

Th polarization is another process that correlates with Ca^2+^ dynamics in T cells after their activation. Upon identical stimulation, Th1 lymphocytes display higher mtCU activity compared with Th2 [68]. Accordingly, Th2 cells possess more efficient mechanisms for cytosolic Ca^2+^ clearance [69]. Finally, differences between Th1, Th2 and Th17 in terms of Ca^2+^ dynamics have been found. Th1 cells display high-amplitude elevations and multiple oscillations after TCR activation, whereas Th2 cells exhibit only a few post-stimulation oscillations and fast recovery to baseline, albeit a significant initial rise in Ca^2+^ level; Th17 lymphocytes show an intermediate pattern with Ca^2+^ response amplitude higher than Th2 but lower than Th1 and Th1-type oscillations [70]. During IL-6-driven Th2 differentiation, increased mitochondrial Ca^2+^ and NCX are required to sustain late NFAT accumulation during activation of CD4^+^ T cells (see below for more details) [71].

## 3. T cell Ca^2+^ dynamics

**Figure 2.**
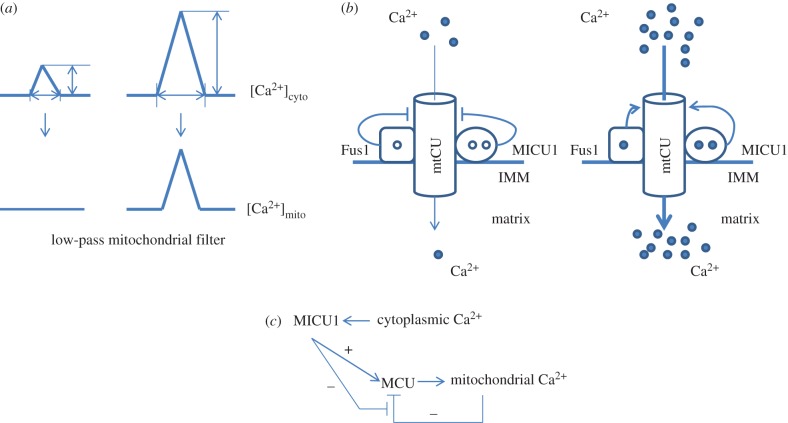
Mitochondrial decoding of cytosolic Ca^2+^ signals. (*a*) Elevations of cytosolic Ca^2+^ with low amplitude and short duration will not produce a significant rise in mitochondrial Ca^2+^, whereas Ca^2+^ signals with sufficient amplitude and duration will increase the Ca^2+^ content of mitochondria. (*b*) Binding of calcium ions with the Ca^2+^-binding protein MICU1 (and possibly with the tumour suppressor Fus1) promotes the opening of the Ca^2+^-permeable channel MCU (pore component of mtCU mechanism) leading to the influx of Ca^2+^ ions into mitochondria (right). At steady state, MICU1 (and probably Fus1) functions as the MCU gatekeeper to prevent basal accumulation of Ca^2+^ (left). (*c*) After Ca^2+^ loading, mitochondrial Ca^2+^ uptake can self-inhibit. The activation of the MCU by MICU1 allows for a regulatory loop preventing MCU self-inhibition, thus prolonging the open state of the channel.

Obviously, the physiological and pathological function of mitochondrial Ca^2+^ transport should be tightly associated with its biochemical and biophysical properties. As mentioned above, the mtCU has a rather low affinity for Ca^2+^ ions, thus setting a high threshold for Ca^2+^ signals in mitochondria [15,22,24,36]. In this way, subthreshold Ca^2+^ signals are not transmitted to—nor affected by—mitochondria [72] (figure 2*a*). From a molecular perspective, this threshold is related to the expression of the MICU1 protein as in its absence mitochondria accumulate Ca^2+^ already at submicromolar content [73–75]. Further, MICU1 amplifies and accelerates Ca^2+^ uptake at higher Ca^2+^ concentration by preventing MCU self-inhibition [74] (figure 2*b*). De la Fuente *et al*. [74] conclude that the ability of MICU1 to lag Ca^2+^ response in mitochondria in time-consuming mode may result in their unresponsiveness to small amplitude and fast Ca^2+^ changes; downregulation of MICU1 would ‘give a green light’ for Ca^2+^ signals with these characteristics to be transmitted to mitochondria [11,74]. Because of this regulation of mtCU by MICU1, Ca^2+^ dependence of mtCU is biphasic: Ca^2+^ release from ER via IP3R triggers mtCU activation, whereas sustained cytosolic Ca^2+^ response suppresses mtCU [23]. Activation occurs on a timescale of approximately 10 s, whereas inactivation takes several minutes to establish [74]. Another player setting the mtCU threshold was identified in our study: a mitochondrial resident tumour suppressor Fus1/Tusc2 possessing Ca^2+^-binding EF-hand motif similar to MICU1 protein [49,76]. In the absence of Fus1, lymphocytes and immortalized epithelial cells demonstrated 1.5 to 2 times greater levels of mitochondrial Ca^2+^ levels than wild-type cells [49] (figure 2*b*).

**Figure 3.**
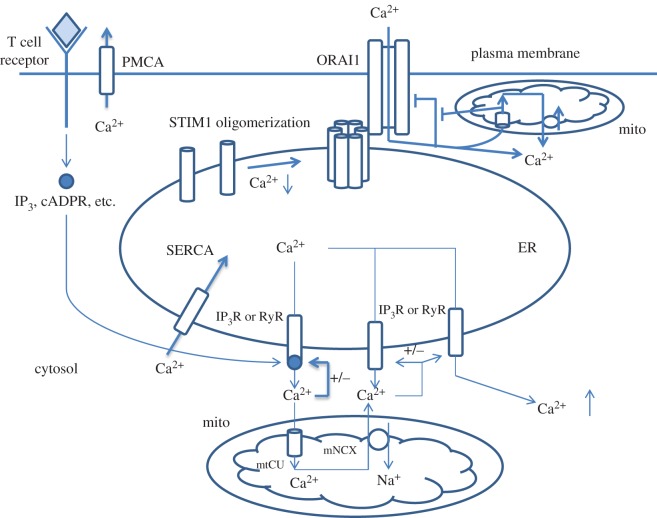
General scheme of store-operated Ca^2+^ entry (SOCE) signalling in non-excitable cells such as T lymphocytes. Activation of plasma membrane receptors (e.g. T cell receptors) leads to the production of second messengers (IP3, cADPR, etc.) involved in Ca^2+^ release from the ER. Ca^2+^ that is released from the ER accumulates in the mitochondria via mitochondrial Ca^2+^ uptake (mtCU) mechanisms. In Ca^2+^-depleted ER, Ca^2+^-binding protein STIM1 changes conformation and oligomerizes; it induces its interaction with the plasma membrane channel ORAI1, the pore-forming subunit of Ca^2+^ release-activated channel (CRAC) and allows the entry of Ca^2+^ ions from extracellular space. ORAI1, IP3Rs and RyRs have the ability to self-inhibit after significant Ca^2+^ rise, which prevents cell from Ca^2+^ overloading. By taking up extra amounts of Ca^2+^ close to Ca^2+^ release channels, mitochondria extend the open state of these three channels. Furthermore, the mNCX provides a positive feedback to ER Ca^2+^ release via activation of IP3Rs and RyRs through local elevations of Ca^2+^ ions at the ER/mitochondria interface.

To better understand how mitochondria shape intracellular Ca^2+^ signals and patterns, we need to review the mechanisms of Ca^2+^ oscillations in non-excitable cells such as T lymphocytes. Non-excitable cells use so-called SOCE and Ca^2+^-induced Ca^2+^ release (CICR) mechanisms to orchestrate Ca^2+^ oscillations [77–79]. Ligation of membrane receptors (e.g. TCR) triggers the formation of the second messenger IP3, which binds its receptor on ER stores leading to the opening of IP3R channels and massive Ca^2+^ release [78] (figure 3). ER possesses transmembrane Ca^2+^ sensor called stromal interaction molecule 1 (STIM1) containing EF-hand domain facing intraluminal space. The Ca^2+^ drop in the ER causes conformational alterations in STIM1 and its oligomerization, resulting in its interaction with the plasmalemmal Orai1 protein, the pore-forming subunit of CRAC, followed by the opening of Orai1 and Ca^2+^ influx into the cell [78] (figure 3). Another mechanism, CICR, maintains Ca^2+^ release from ER store: after reaching the threshold level, locally increased Ca^2+^ sensitizes IP3R and RyR to opening and propagation of Ca^2+^ waves along the ER [77,80,81]. It is noteworthy that both channels, CRAC and IP3R, create local Ca^2+^ domains with high Ca^2+^ concentration, which can autoinhibit channels [82,83]. This self-suppression is prevented by mtCU restricting local Ca^2+^ concentration [79,84] (figure 3). Finally, plasma membrane (PMCA) and ER store (SERCA) Ca^2+^ ATPases contribute to decay phase of oscillations and terminate Ca^2+^ signal; lower affinity of Ca^2+^ ATPases to Ca^2+^ and slower rates, compared with IP3R and RyR, allow them to enter the process of Ca^2+^ signal propagation later than Ca^2+^ channels, thus allowing Ca^2+^ response to develop [79].

A mathematical model shows that the optimal distance between the ER and mitochondria should be 30–85 nm; when it is less, mitochondria prevent positive feedback of low Ca^2+^ on IP3R, whereas above 30–85 nm, mitochondria take up Ca^2+^ and preserve IP3R from Ca^2+^-induced inhibition [85]. Moreover, the ability of mitochondria to accumulate Ca^2+^ released from the ER store indirectly causes STIM1 oligomerization and activation of the CRAC current [86,87]. Finally, accumulated Ca^2+^ is released from mitochondria by the mNCX and owing to the proximity of the ER, mitochondria create local Ca^2+^ domains, which sensitize IP3R and RyR to Ca^2+^ release, thus triggering CICR [88,89]. It is important that both the mtCU and the mNCX have an influence on the shape and the dynamics of regenerative Ca^2+^ oscillations [20,89,90]. Finally, the NCLX is a key for refuelling the ER with Ca^2+^ ions in B cells, whereas NCLX-mediated Ca^2+^ mitochondria/ER recycling is central in antigen stimulation of B cells [91]. Collectively, these data indicate that mitochondria provide cellular Ca^2+^ influx mechanisms with positive feedback loops, which are important for generation of digital signals (discussed below).

Oscillations in cytosolic Ca^2+^ translate into transcriptional changes based on frequency, amplitude and spatial decoding of Ca^2+^ signals [79,92,93]. Classical examples are NFAT and NFκB transcriptional factors dependent on the frequency of Ca^2+^ oscillations: NFAT requires more frequent oscillations (approx. every 6 min) to maintain a transcriptionally active state, whereas NFκB needs less frequent fluctuations (every 30 min) [94]. This is explained by the mechanism of activation and cytosol/nuclear shuttling of NFAT and NFκB. Ca^2+^ activates the CaM-dependent phosphatase CaN, leading to dephosphorylation of inactive NFAT followed by its translocation into the nucleus, whereas re-phosphorylation of NFAT promotes its return into the cytosolic compartment. Thus, rapid periodic oscillations are required for NFAT activation state via maintenance of its dephosphorylated state [95]. At the same time, the active state of NFκB is defined by Ca^2+^-induced proteolysis of its inhibitor, IkB, and relocation of NFκB from the nucleus into the cytosol, which requires binding of NFκB with IkB. Re-synthesis of IkB is counterbalanced by its Ca^2+^-triggered proteolysis; thereby, Ca^2+^ fluctuations with a period of 30 min are able to maintain NFκB in the activated state [95].

Coming back to the initial question of why T cells use mitochondria to make decisions involving Ca^2+^ signalling, we assume a simple concept formulated in the next way: if mitochondria are used for this process, it means that they meet requirements/criteria for the computational tasks of signal processing they are provided with. However, how do T cells decode the information transferred from the APC to T cells' nuclei, and help in computing cell decisions? To respond to this question, we have to take into consideration specificity of T cell activation (i.e. how T lymphocytes distinguish specific antigenic peptides from non-specific at the levels of information processing and decision-making). Altan-Bonnet & Germain [96] showed that both types of peptides, specific and non-specific, bind TCR and induce tyrosine kinase cascade together with the negative feedback loop, including tyrosine phosphatase SHP-1 dephosphorylating TCR targets and overall signal dampening. At the same time, only agonistic peptides stimulate an MAPK module via a positive feedback loop originating from TCR triggering. This effect is based on the longer lifetime of TCR/MHC/peptide complexes characteristic for specific interaction [96]. Negative (SHP-1) and positive (MAPK) feedback loops are activated consequently: SHP-1 becomes activated after short TCR/MHC interaction, whereas MAPK activation needs a longer time for TCR engagement by pMHC. That means that T cells discriminate specific recognition from a non-specific one by a time lag in signalling: if the pMHC complex does not match the target TCR, then downstream signalling will not activate MAPK, because SHP-1 will barely suppress TCR downstream signals; on the contrary, a longer interaction of pMHC with TCR will result in the activation of MAPK and the consequent maintenance of the T cell activation programme [96]. This important role of time lags in T cells reading Ca^2+^ signals has been demonstrated in another work focused on the signal discrimination by autocorrelation of detrended time series [97]. Autocorrelation (serial correlation) is a mathematical function allowing the discovery of patterns in signal dynamics, based on the quantification of the level of self-similarity of a signal observed at different time intervals. Detrending of time series consists of removing a time-delayed tendency of time series, resulting in signal differencing/rationing. Translating to cellular processes, this work [97] proposed that oscillation frequency-dependent shuttling dynamics of NFAT and NFκB provide cells with detrending procedure/computing similarly to described above. Further, activation of NFAT- and NFκB-dependent transcription starts after overcoming threshold in average intracellular Ca^2+^ concentration leading to filtering out non-functional noise from functional data [94,97].

Thus, cellular systems involved in signal reading should demonstrate specific features such as filtering properties (thresholding) and memory in signal dynamics to compute difference between two time points (series). The importance of signal memory has been demonstrated *in vivo* for tumour rejection: prolonged activation of TCR leads to sustained nuclear NFAT accumulation and transcription of *IFNg* gene, whereas short-term transitory stimulation is accompanied by only transitory NFAT nuclear translocation and *Egr2* gene upregulation inducing CD8^+^ T cell tolerance [98].

At this point, we can already draw some conclusions about the involvement of mitochondria in Ca^2+^ signalling from the perspective of information processing. Mitochondria have (i) a threshold for Ca^2+^ signals (MICU1, Fus1) [49,73–76], and (ii) a ‘lagging’ mechanism between onsets of cytosolic and mitochondrial Ca^2+^ elevations, which allow to process the incoming information (input) by analysing both the amplitude and the duration of Ca^2+^ responses (MICU1-mediated block) [74,99]. This delay is due to the overall slower Ca^2+^ dynamics in mitochondria induced in large part by the large Ca^2+^ buffering capacity of this organelle. Also, (iii) MICU1 removes self-inhibition (negative feedback loop) of mtCU [74], and (iv) Ca^2+^ removal from mitochondria through the mNCX triggers Ca^2+^ oscillations, after the opening of mtCU and accumulation of mitochondrial Ca^2+^ (positive feedback loop) [89,90]. The importance of the coordination between mtCU and mNCX mechanisms in the general scheme of cellular Ca^2+^ signalling is supported by the fact that MICU1 and Fus1 are both involved in the control of mNCX besides their effect on mtCU [49,73,76]. Thus, mitochondria ‘send’ a triggering signal to ER store, but it is possible after filling the mitochondrial compartment with Ca^2+^ [100]. In turn, the input signal should have enough strength and duration, otherwise, it will be filtered out by mitochondria [72]. Such a complex character of the transport mechanism of mitochondrial Ca^2+^ might be resolved if one considers that mitochondrial Ca^2+^ transport is a part of a molecular proportional−integral−derivative (PID) controller often used in engineering applications. In the following, we analyse mitochondria from this prospective.

## 4. Mitochondria as a module of cellular proportional–integral–derivative controller

**Figure 4.**
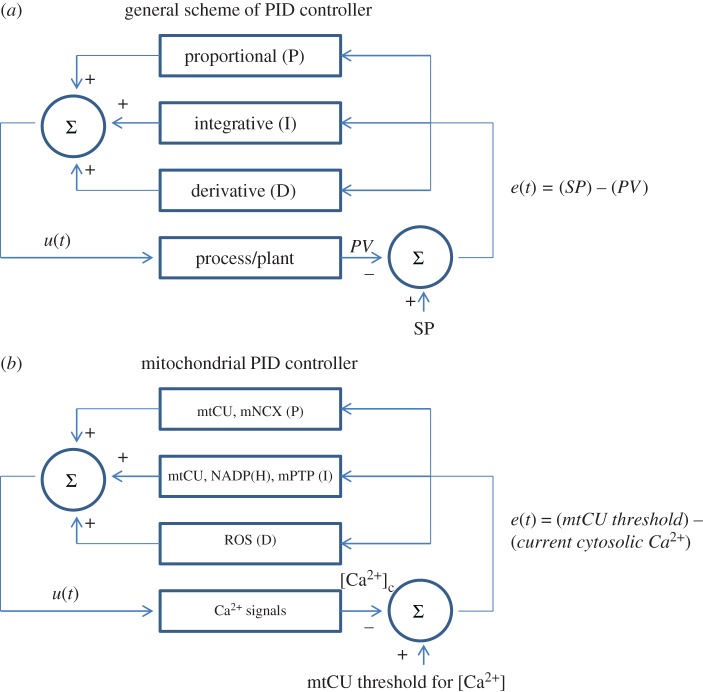
Mitochondrial Ca^2+^ transport is analogous to a proportional–integral–derivative (PID) controller. (*a*) Block diagram of a PID controller, an engineering application that uses feedbacks to continuously generate an output signal in a dynamic system until it reaches a well-defined reference value. An error *e*(*t*) is generated continuously by comparing the SP with the current value of the parameter controlled by the system. Three elements within the PID controller produce three modes of output: proportional (P), integrative (I) and derivative (D) functions of *e*(*t*). The outcome of the three terms is then summed (∑) to give a system's output. (*b*) Mitochondrial Ca^2+^ transport as the PID controller for Ca^2+^ oscillations. Error signal (*e*(*t*)) represents the difference between mtCU threshold (*SP*) and current Ca^2+^ concentration (*PV*). Mitochondrial Ca^2+^ transport and associated metabolic processes display the same modes as those of the PID controller elements. Mitochondrial Ca^2+^ uptake (mtCU) and export (mNCX) mechanisms are proportional (P) to the Ca^2+^ loading of mitochondria, whereas mtCU, Ca^2+^-dependent NADP(H) response and the PTP in its low-conductance mode represent the I element. Finally, mitochondrial ROS production corresponds to the D element.

PID controllers are sensitive to the magnitude of input signals and correct it in the course of time according to a controller function *u*(*t*) defined as
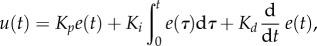
where *u*(*t*) is the controller output, *Kpe*(*t*) is the proportional function, 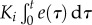 is the integral term and 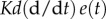 is the differential component of the PID controller [101]. Error *e*(*t*) represents the difference (*e* *=* SP − PV) between a well-defined set point (SP) and the current value of a given variable (process variable, PV; cytosolic Ca^2+^ concentration in this example). The PID controller computes the output signal based on the sum of the proportional (P), integral (I) and derivative (D) terms (figure 4*a*). The proportional term helps to adapt the process variable proportionally to the error. The derivative term responds to the error depending on how fast the error is approaching zero. Finally, the integral term operates based on the accumulated error and thus set PV proportionally to past errors (figure 4*a*). If the PID controller overshoots the SP, the output signal would oscillate around the SP with increasing, decreasing or constant amplitude, thereby reflecting the system stability [101].

PID controllers are widely used in industry for regulation of temperature, speed, pressure, flow, etc. [101]. The erythropoietin system represents an example of molecular PID controller in living systems, where an optimal O_2_ concentration acts as the reference SP, whereas the signal error (*e*(*t*)) is erythropoietin (Epo) secretion induced via HIF1/HIF2 activation by low oxygen content [102]. Early erythroblasts form a PID controller based on the findings that EPO (i) proportionally downregulates Bim and Fas, which promote apoptosis of these cells (proportional function), and (ii) increases in a digital mode the expression of Bcl-xL, a pro-survival Bcl-2 family member (derivative function). It results in erythroblast expansion during erythropoietic stress *in vivo*. However, what is an integrative component is still not defined although it might be a self-renewal process [102]. The resulting output will be a change in red cell mass and amount of tissue O_2_. The difference between current O_2_ concentration and optimal (set) O_2_ content defines the amount of Epo secretion [102]. On a molecular level, the PI controller model has been recently applied for mammalian central carbon metabolic pathway in normal and cancer cells [103]. At the organism level, the PI controller was proposed for homeostatic mechanisms responsible for inflammation and glucose level control [104]. Undoubtedly, in the future, other biological PI(D) controllers will be described.

Although speculative, it seems that mitochondria may act as part of a PID controller for T cell activation through the regulation of cellular Ca^2+^. The PV would be a given value of cytosolic Ca^2+^, which is continuously adjusted to the SP by the appropriate Ca^2+^ fluxes between the cytosol and the mitochondria. Which are the processes that might be considered as proportional, integral and derivative terms? Most probably, transmitochondrial Ca^2+^ transport (mtCU + mNCX) represents the *proportional* element of PID controller. Mitochondria proportionally increase Ca^2+^ uptake after an increase in cell Ca^2+^ load [105]. The ability of mtCU to respond proportionally to a cytosolic Ca^2+^ increase is apparently based on the mtCU cooperativity determined by the presence of two Ca^2+^-binding sites on the MICU1 protein, the regulatory subunit of MCU [73], and on the presence of Ca^2+^ buffers (e.g. Pi) in mitochondrial matrix [31,106]. The Hill coefficient (defining the degree of cooperativity) for mtCU has a value of 2.4 [105]; in the classical example of cooperative binding of oxygen with haemoglobin, the Hill coefficient is approximately 2.3–3 [107]. Pi buffer helps to maintain ΔΨm polarized after initial Ca^2+^ uptake, thereby helping mitochondria to take up Ca^2+^ ions. It is important to note that the rate of dynamic change in ΔΨm is proportional to the rate of gain in Ca^2+^ uptake [106].

There is another, more indirect way by which the mtCU produces an output value that is proportional to the current error value (i.e. the current cytosolic Ca^2+^ concentration). The cytosolic Ca^2+^ response including Ca^2+^ oscillations indeed relies on the process of STIM1-dependent oligomerization and activation of the SOCE Ca^2+^ pathway. In turn, STIM1 oligomerization is proportional to the depletion of the endoplasmic Ca^2+^ store, which is indirectly controlled by the mtCU through both the MCU and UCP2 [86]. Moreover, the magnitudes of the mitochondrial and cytosolic Ca^2+^ responses directly correlate [86], thus demonstrating that the gains in cytosolic and mitochondrial Ca^2+^ are proportional. This effect depends on mtCU activity. Finally, gradual dissipation of ΔΨm, the driving force for Ca^2+^ exchanges between the cytosol and mitochondria, proportionally diminishes Ca^2+^ oscillations in mast cell line RBL-1 [90]. Besides the mtCU, the mNCX might also contribute to shaping the P-term of PID controller (figure 4*b*). Indeed, the significance of mNCX for cytosolic Ca^2+^ oscillations is well documented and based on the ability of the mNCX to fuel the ER store with the amount of Ca^2+^ ions necessary to trigger CICR and the accompanying Ca^2+^ oscillations [88,89].

Another term of the mitochondrial PID controller, *integrative*, might involve some components responsible for Ca^2+^ transport and accumulation that demonstrate an ability to summarize incoming signals. In particular, mtCU cooperatively with Pi buffers integrates repetitive cytosolic Ca^2+^ signals resulting in a frequency-dependent net accumulation of Ca^2+^ ions in mitochondria [105,108] (figure 4*b*). As mentioned above, Ca^2+^ activates the TCA cycle and production of NAD(P)H [43]. The redox response usually recovers to basal levels much later than the Ca^2+^ signal triggering the changes in NAD(P)H production [72], although the mNCX significantly accelerates the return of the redox state to its steady values [109–111]. Furthermore, an increase in the frequency of Ca^2+^ signals leads to a larger NAD(P) reduction even if the decline in NADP(H) from a previous signal still propagates, thereby summarizing responses from few inputs [72]. Thereby, the metabolic status of mitochondria might play the role of integrator in the proposed mitochondrial PID controller. Finally, Ca^2+^ can leave mitochondria via the mPTP in its low-conductance mode, a process that also shows the characteristics of an integrative element in physiological conditions. Fast rates of repeated Ca^2+^ stimulation favour low-conductance flickering of the mPTP (permeability < 300 Da) and consequent Ca^2+^ release [112]. MPTP opening is dependent on pH and ΔΨm [112,113], two processes that depend on mitochondrial Ca^2+^ accumulation after repeated cytosolic Ca^2+^ stimuli.

**Figure 5.**
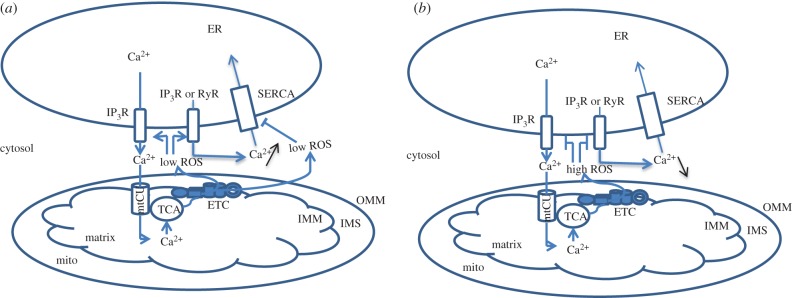
Dual regulation of Ca^2+^ signalling by ROS. At low concentration (*a*), ROS, stimulated by Ca^2+^ accumulation in mitochondria and stimulation of TCA and ETC, activate Ca^2+^ release from the ER and inhibit ER ATPases thereby promoting a Ca^2+^ response in the cell. At high concentration (*b*), ROS inhibit Ca^2+^ channels, thus dampening further expansion of the Ca^2+^ response.

The *derivative* element of the PID controller anticipates the system behaviour based on the calculation of the slope of the error with respect to time. We propose here that mitochondrial ROS production might represent the D term of the mitochondrial PID controller. Treatment of cells with mitochondria-targeted antioxidants abolishes cytosolic Ca^2+^ oscillations induced by physiological stimulation [114–116]. This effect is based on the ability of ROS to oxidize protein thiols and inhibit PMCA and SERCA while sensitizing RyR and IP3R to opening and Ca^2+^ release [114,115]. It is important that the effect of ROS on ER Ca^2+^ release channels is dual: RyR opens at moderate concentrations of ROS but becomes inhibited at high ROS concentration [117]. ROS accumulation in mitochondria is balanced by its production in the ETC and utilization by antioxidant systems (SOD, catalase, peroxidase) [118]. Production of ROS is stimulated by elevation in matrix Ca^2+^ leading to an increase in the electron flux of the ETC [119]. It has been shown that mitochondria-derived ROS regulate cytosolic Ca^2+^ sparks in a time/history-dependent manner. During the first phase, ROS-sensitized ER channels release Ca^2+^ (figure 5*a*). Emptying Ca^2+^ ER store is accompanied by expansion of mitochondrial ROS production. In the second phase, high concentrations of ROS significantly depress Ca^2+^ sparks [116] (figure 5*b*). Thus, the level of Ca^2+^ is dynamically regulated by the rate of the mitochondrial ROS changes. Besides, the buffering of ROS in mitochondria is tightly connected to the mitochondrial NADH pool as alterations of only one parameter in the antioxidant system (e.g. superoxide dismutase concentration) can significantly change the amplitude and the frequency of mitochondrial oscillations (e.g. ROS outcome of mitochondrial oscillator) [118,120,121] (figure 4*b*).

ROS also play a role in the dynamic and concentration-dependent regulation of glucose metabolism that couples glycolysis and ETC fuelling with reducing equivalents. Indeed, short exposure of cells to ROS triggers glucose uptake, its conversion into pyruvate and hyperpolarization of the inner mitochondrial membrane. By contrast, prolonged presentation of ROS leads to downregulation of glucose transport in order to prevent excessive feeding of ETC with electron-donor molecules [122]. Thus, ROS potentially couples Ca^2+^ signalling, ETC activation and metabolic reprograming (in particular, alterations in glucose transport) in a manner that depends on the temporal changes in ROS production associated with T cell development.

PID controllers have an important limitation: they have a low capability to minimize noise. In engineering, to remove noise in the system using PID-based control system, low-pass filters (e.g. Kalman filter) for feedback response are used [101]. In the case of mitochondria, such a role might be ascribed to the MICU1 protein, which has two important characteristics as a low-pass filter: (i) MICU1 filters out signals with low amplitude [72]; (ii) the protein removes MCU block in a time-dependent mode proportionally to the strength of cytosolic Ca^2+^ signal [74].

After performing the analogue computational procedures, the PID controller combines outcomes from all three terms (proportional, integrative and derivative) and sends a final (summarized) decision to elements of the driving process [101]. How does the mitochondrial PID controller compute the resulting outcome based on its P, I and D terms? If we imagine that individual cells would experience sustained Ca^2+^ signals with high amplitude, then we could expect that mitochondria will be proportionally accumulating Ca^2+^ with equal emptying of the ER store as the mNCX would provide the IP3R and RyR with enough Ca^2+^ ions to trigger their opening. That would favour CRAC channels in an active state. Concurrently, Ca^2+^ response will be tuned out by the amount of ROS and availability of antioxidant buffers. Thus, the resulting response will be directed by the summation of PID controller terms. If the value of any parameter (beginning with Ca^2+^ signal length/amplitude/frequency and ending with NAD(P)H concentration) is changed, then the PID controller outcome will be adjusted according to these modifications. In our case, the resulting process might involve Ca^2+^ signalling and Ca^2+^-dependent signal transduction systems such as NFAT cytosol/nuclear shuttling. For example, during Ca^2+^ responses, the treatment of cells with pro-oxidant (analogue of excessive mitoROS production) leads to the CaN inhibition and the dampening of Ca^2+^-induced NFAT nuclear translocation, whereas the prevention of oxidative stress (analogue of high NAD(P)H production) suppresses this effect [123].

Before further discussion, we return to the properties of the mtCU and remind that (i) the mtCU acts as a low-pass filter, which has a high threshold [15,22–24,99]; (ii) the dissipation of ΔΨm proportionally decreases Ca^2+^ oscillations [90]. We propose that the Ca^2+^ threshold for mtCU activation might serve as an SP for Ca^2+^ oscillations. Indeed, Ca^2+^ oscillations would be maintained until the error would reach zero; in other words, when SP = PV and *e* = SP − PV = 0. However, while PV is greater than SP, the Ca^2+^ system will be oscillating owing to the difference between the cytosolic Ca^2+^ concentration and the threshold for mtCU opening. It is noteworthy that these two parameters are coupled via the mtCU-dependence of STIM1 oligomerization and further interaction with CRAC1, the pore unit of SOCE currents [86]. Mitochondria indeed take up Ca^2+^ from the ER store, thus emptying it and allowing the ER Ca^2+^ level to drop below the *K*_d_ for Ca^2+^ binding to the EF-hand of STIM1, thereby leading to its oligomerization [84,124].

As mentioned above, if the PID controller overshoots the SP, the output signal will oscillate around the SP [101]. In the framework of the dynamics of nonlinear systems, oscillations (cellular Ca^2+^ oscillations, mitochondrial oscillations, etc.) arise at special points called Hopf bifurcations [120,125,126]. The bifurcation point represents a point in the parameter space where the system changes behaviour in a qualitative mode (e.g. from a steady state to self-sustained oscillations) [126]. In this regard, *e* = SP − PV = 0 would be analogous to the bifurcation point between these two states.

The SP, PV and *e* are flexible as cells can use different strategies to shape cellular Ca^2+^ signals through post-translation or transcriptional regulation of proteins involved in ER/mitochondria Ca^2+^ crosstalk:

(1) reduction of Ca^2+^ currents by depolarization of cellular membrane potential, downregulation of IP3R or RyR, modified efflux of Ca^2+^ by Ca^2+^ ATPase (PMCA or SERCA);
(2) changes in sensitivity of the mtCU to Ca^2+^ (MICU1, Fus1), mitochondrial Ca^2+^ export or expression of its components (e.g. NCLX1), ΔΨm increase or decrease; and
(3) alterations in the distance between the ER and mitochondria (e.g. up/downregulation of mitofusins, Grp75/HSPA9).

How can T cells use these strategies in order to regulate their fate? IP3R-mediated Ca^2+^ release is important for the initial production of cytokines [127] but not for late expression of cytokines by activated CD4^+^ T cells [128] where mitochondria are the most plausible source for cellular Ca^2+^ exchange [71]. It is well documented that T cells use two major types of K^+^ channels: voltage-gated Kv1.3 and Ca^2+^/calmodulin(CaM)-regulated KCa3.1. The Kv1.3 channel has a voltage sensor located in the fourth transmembrane domain containing four arginine residues, whereas the C-terminus of KCa3.1 channel bears intracellular CaM-binding motif and senses Ca^2+^ elevations [129]. Naive T cells express mostly Kv1.3 channels, whereas T cells upregulate KCa3.1 channels after TCR-triggered activation [129]. There is also a difference in Kv1.3 and KCa3.1 usage described for central-memory T(CM) and effector-memory T(EM) cells: upon activation, T(CM) lymphocytes display dependence on KCa3.1 channels, whereas T(EM) rely on Kv1.3. It is believed that in resting T cells, the opening of Kv1.3 channels repolarizes membrane potential after transient depolarization, whereas blocking a Kv1.3 channel leads to diminished Ca^2+^ currents owing to a decrease in the driving force for Ca^2+^ [130]. An increase in the intensity and the density of Ca^2+^ currents in activated T cells is accompanied by an upregulation of KCa3.1 channels, which hyperpolarizes the membrane potential and maintains CRAC influx [131–134]. The Th1 subset demonstrates higher KCa^2+^ currents compared with Th2, which is translated into stronger and sustained Ca^2+^ signals in Th1 cells [69]. In contrast, Th2 have an elevated level of Trpm4, a Ca^2+^-dependent Na^+^ channel; opening of Trpm4 leads to depolarization of the membrane potential and reduced Ca^2+^ oscillation patterns [135]. Thus, changes in expression of K^+^, Ca^2+^ or Na^+^ channels pursue the goal of fine-tuning Ca^2+^ responses according to a programme dictated by different steps in T cell life. The role of mitochondria in these processes might be to set the parameters responsible for the spatio-temporal Ca^2+^ patterns such as oscillations.

## 5. Analogue and digital signalling in T lymphocytes

Another important aspect of T lymphocyte life is the transduction of stimuli with different intensity into analogue (gradual) or digital (binary) outcomes in order to adequately respond to environmental signals. An early study demonstrated that in T cells, parameters of Ca^2+^ mobilization (magnitude, increase rate, frequency of oscillations, etc.) after specific stimulation display incremental characteristics when increasing the amount of MHC/peptide molecules [136]. However, a recent investigation showed that a single peptide/MHC complex can initiate the response of CD4^+^ T cell and that additional pMHC molecules do not increase the secretion of effector cytokines (such as IL-2 or TNFa), demonstrating an ‘all or none’ type of response [137]. The follow-up of the secretion of Th2 cytokine, IL-4, strictly requires the presence of NFAT in the nucleus and analogue differences in TCR triggering are converted into digital outcome of NFAT nuclear translocation [138]. It is noteworthy that activation of NFAT itself is a digital process because it requires the complete—highly cooperative—dephosphorylation by CaN of 13 serine residues, which hide the nuclear localization signal segment inside the protein [139]. Moreover, CRAC-driven activation of NFAT is also described as an ‘all or nothing’ response: opening of CRAC channels creates local Ca^2+^ domains, which cause CaN activation if they reach the threshold Ca^2+^ concentration; CaN-dependent dephosphorylation of NFAT overcomes another threshold set by the highly phosphorylated state of inactive NFAT [140]. As described above, TCR specificity is dependent on a digital ERK activation based on the high amplification of the MAPK module [96]. At the same time, MAPK responds gradually to stimulation of Jurkat cells with SDF-1, reflecting plasticity in the reaction of T cells to chemokine gradients [141]. In addition, the activation of NFκB showed analogue [138] and digital [142] modes after gradual T cell stimulation.

**Figure 6.**
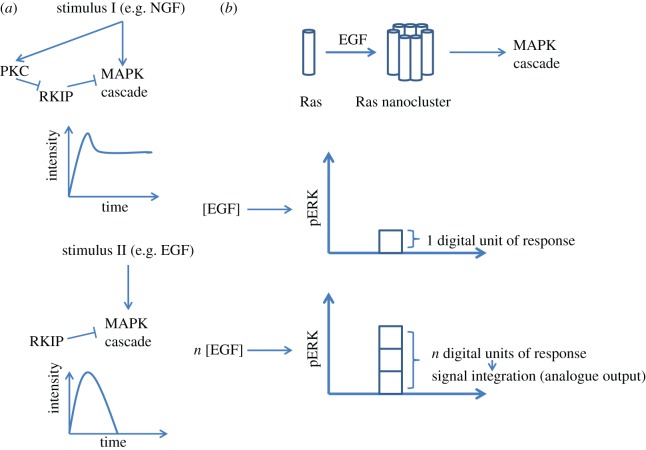
Digital and analogue activation of the MAPK cascade. (*a*) Top: stimulus I leads to activation of MAPK cascade together with PKC, which keeps MAPK stimulated over a long time as RKIP, a suppressor of MAPK signalling, is inhibited. This creates a positive feedback forward loop (digital mode). Bottom: stimulus II does not activate PKC, which results in the decrease of MAPK cascade activation with time; the strength of such a response will depend on the amount of stimulus II (analogue mode). (*b*) Top: upon EGF stimulation, Ras molecules form nanoclusters, which stimulate MAPK cascade. Bottom graphs: each nanocluster gives 1 digital unit of ERK activation while combining responses induced by distinct nanoclusters leads to a higher overall response. The level of response is determined by the concentration of EGF capable to stimulate the formation of more Ras nanoclusters, via binding to the receptor (analogue output).

The importance of digital and gradual signals has been demonstrated for the MAPK module activation in PC-12 cells. Endothelial (EGF) and neuronal (NGF) growth factors both activate Erk1/2 MAPK, but with different modalities: gradual for EGF and digital for NGF [143]. Functionally, EGF stimulates proliferation, whereas NGF directs cells toward differentiation. The mechanism of rewiring of the MAPK pathway from analogue to digital mode relies on a positive feedback loop: NGF stimulates PKC, which in turn phosphorylates and inhibits Raf kinase inhibitory protein (RKIP), thus keeping the MAPK phosphorylation cascade turned on (figure 6*a*, stimulus I). In the case of EGF, this mechanism is lacking as only a negative feedback loop is present, which implies that the strength of signal transduction is determined exclusively by the amount of ligand (input signal; figure 6*a*, stimulus II) [143]. Another example that represents a high-fidelity analogue–digital–analogue converter is based on Ras nanoclusters [144]. Ras is a small GTPase protein anchored in cellular membranes, which participates in signal transduction from membrane receptors to the MAPK module. After ligand–receptor binding, Ras coordinately aggregates into nanoclusters with about seven molecules per group and a lifetime of about 0.5 s. Then, Ras clusters send a downstream signal towards ERK1/2 via interaction with the Raf kinase (figure 6*b*, top). Ras nanocluster formation is digital in its nature, because GTPase molecules start clustering after reaching a given threshold. Below this threshold, Ras signalling does not provide any significant output. The amount of clusters, however, depends on the ligand concentration and displays analogue characteristics when Ras is above the threshold. The signal output then rises proportionally to the input. This process is called signal integration and is necessary for high-fidelity signal transduction when the output (ERK activation) should precisely reflect the input (ligand concentration) signal (figure 6*b*, bottom graphs) [144,145].

**Figure 7.**
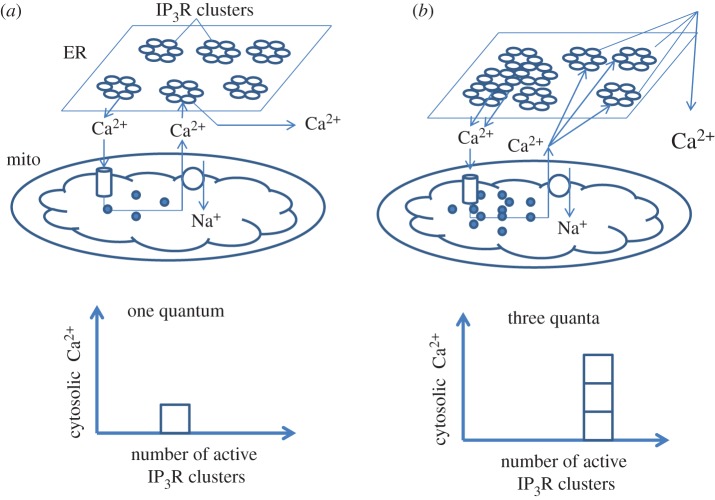
The possible role of mitochondria in gradual transformation of Ca^2+^ signals. (*a*) After an IP3-induced Ca^2+^ release of low magnitude (frequency, duration or amplitude), Ca^2+^ recycling maintained by mitochondria will lead to a low activation of IP3Rs and RyRs (1 relative quantum of signal). (*b*) Higher amplitude of Ca^2+^ release from the ER will result in larger Ca^2+^ released from mitochondria and in a proportional gain of the overall Ca^2+^ response (three relative quanta). Thus, the integration by mitochondria of discrete Ca^2+^ pulses from individual clusters of channels in the ER might be considered as a digital–analogue conversion. As the output of each ER storage channel (IP3Rs, RyRs) is dumped into ER/mitochondria cleft, the digital pulses are summed to give a final analogue output expressed by the activity of transcription factors (i.e. NFAT, NFκB). The existence of such biological circuits is necessary for the generation of high-fidelity signals.

For T lymphocytes, fine-tuning of NFAT dynamics requires high-fidelity signal transduction owing to the extreme importance of NFAT-mediated transcription in cell decisions. Disturbances in this process result in severe pathology [146–148]. As an example, the length of the NFAT half-life in the nucleus defines Th differentiation: after similar strength of stimulus, NFAT is present in the nucleus much longer in Th1 and Th17 when compared with Th2 cells [70,149,150]. NFAT nuclear accumulation and transcriptional regulation demonstrate signs of hysteresis or molecular memory when the system continues producing output signals even when the input trigger is no longer active [98,151]. Indeed, NFAT upregulates its targets even when receptor activation is gone. This process is based on the binding of CaN with NFAT in the nucleus; propagation of Ca^2+^ waves into the nucleus (most probably from perinuclear mitochondria) leads to strong association of both proteins, preventing NFAT from re-phosphorylation and further Crm1-mediated nuclear export [152]. At the same time, sustained dynamics of NFAT nuclear accumulation provides evidences of the presence of a positive feedback loop similar to the MAPK module described above. Moreover, NFAT nuclear retention reflects the longevity of the Ca^2+^ response, thereby exhibiting graded mode. This inter-relationship is determined by the ability of mitochondria to slowly release Ca^2+^ via the mNCX after its accumulation during cytosolic elevations in sensory neurons [123] and CD4^+^ T cells [71]. It is known that the close proximity of mitochondria with ER store allows the mNCX to activate IP3R and RyR owing to the local increase of Ca^2+^ concentration [88,89]. Although the mechanism of NFAT activation and translocation has a digital nature [139,153], transcription factor retention in the nucleus has more analogue characteristics although the curve of NFAT/Ca^2+^ response dependence is very steep and close to a switch-like response [123]. We propose that the ability of NFAT to titrate the duration of the Ca^2+^ responses is very important for its biological effects, because NFAT can trigger different transcription programmes depending on the nuclear lifetime (e.g. activation versus tolerance) [148,154]. The process of mNCX-controlled nuclear NFAT retention reminds us of the signal integration for Ras clusters described above. Indeed, IP3Rs also form dynamic clusters, which are responsible for elementary Ca^2+^ events [81,155] (figure 7). IP3Rs form single quantum (binary) circuits and summation of Ca^2+^ release quanta gives graded response depending on agonist concentration at submaximal doses [156–159]. We assume that mitochondria in this case could be considered as integrating detectors owing to their ability to accumulate Ca^2+^ and release it during the plateau phase following the peak (figure 7), thereby maintaining NFAT in an ‘on’ state in the nucleus. Furthermore, mitochondria are involved in CRAC-driven NFAT activation, which has a digital nature as described above [140]. Taken together, mitochondria might represent a domain involved in analogue–digital and digital–analogue conversions. As mentioned above, cells use this type of system when high fidelity of signal transduction is necessary [144,145].

**Figure 8.**
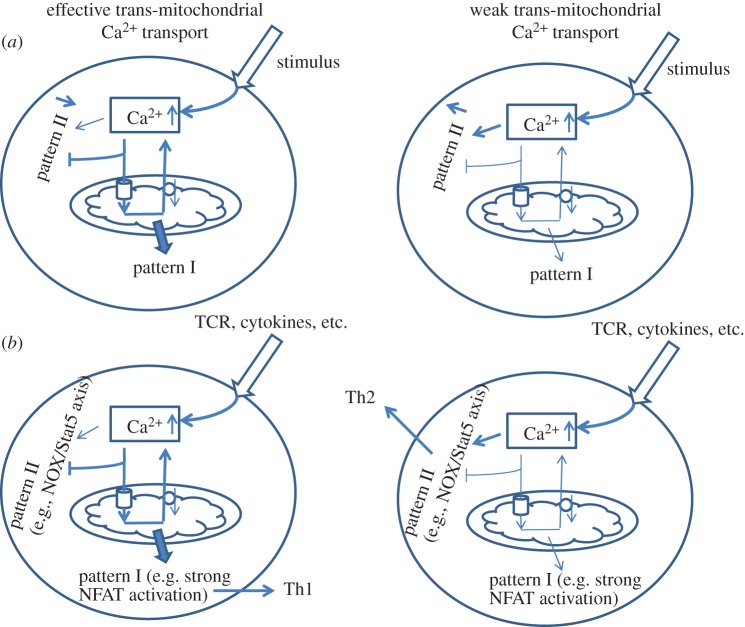
Rewiring of the Ca^2+^-signalling pathways triggering different cellular programmes. (*a*) General scheme of rewiring dependent on transmitochondrial Ca^2+^ transport. If the mtCU and the mNCX are active, elevated Ca^2+^ ions undergo periodic fluctuations and stimulate Ca^2+^ oscillation-dependent proteins (pattern I), whereas cytosolic Ca^2+^ retention will keep these targets inactive (pattern II; left side). Deficiency in mitochondrial transport will favour stimulation of pattern II at the expense of pattern I (right side). (*b*) Proposed scheme of rewiring in Th differentiation. Intense transmitochondrial Ca^2+^ transport leads to strong NFAT activation and bias towards Th1 lymphocytes and suppression of Th2 stemming (left side), whereas diminished mtCU in T cells will be accompanied by a PKC-dependent stimulation of NADPH oxidase, which requires accumulation of intracellular Ca^2+^ leading to Th2 differentiation (right side).

We now briefly review possible mechanisms related to the involvement of mitochondria in the rewiring of the signalling pathways, in order to switch between the various T cell programmes. To achieve this aim, we need to imagine a hypothetical stimulus S, which in a simple assumption activates two signalling pathways Y1 and Y2 via competing sensors X1 and X2, respectively. Both sensors are specific to the mediator Z, but possess different activation parameters (affinity, kinetics, etc.). If the level of Z, reaches the threshold parameters for X2, then the latter will buffer mediator Z and occlude it from interaction with X1, thus keeping pathway Y1 inactive (figure 8*a*). At the same time, if the level of Z is below threshold parameters for X2, then stimulus S will favour activation of sensor X1 via mediator Z, resulting in activation of pathway Y1 (figure 8*a*). Applying this assumption to mitochondria, mtCU opening and Ca^2+^ uptake can limit availability of Ca^2+^ ions to other Ca^2+^-dependent targets. Thus, Akt/PKB-mediated survival requires the release of mitochondrial Ca^2+^, and in the absence of mNCX, the apoptotic programme is triggered [64]. Short-term translocation of NFAT in the nucleus induces a tolerance programme, whereas prolonged retention upregulates the activation programme [98]. Sustained residence of NFAT in the nucleus is strictly necessary for IL-4 synthesis [138]. As described above, EGFR and NGFR can rewire the MAPK module depending on activation of PKC involving a positive feedback loop [143]. It is known that in non-polarized conditions (PMA/ionomycin, CD3/CD28), T cells mostly favour Th1 phenotype with prevalent secretion of IFNg [160–162]. Common features of these responses are high-amplitude and long-term activation of Ca^2+^ responses, which correlate with the requirements for Th1 differentiation [69,70]. At the same time, suppression of transmitochondrial Ca^2+^ transport would lead to different patterns of Ca^2+^ signalling, including lack of regenerative Ca^2+^ oscillations [89,90]. Indeed, blocking the mtCU in human lymphoblasts upregulates the NADPH oxidase, which normally requires Ca^2+^-dependent PKC activation [65]. In turn, NADPH oxidase 5 (NOX5)-deficient T cells show under-regulated phospho-Stat5 and diminished Th2 differentiation [163] (figure 8*b*). Thus, the threshold for mtCU opening might serve as a switch for rewiring between signalling pathways responsible for different T cell programmes.

## 6. Concluding remarks

In this article, we have attempted to characterize mitochondrial Ca^2+^ transport as an engineering application with capability to perform computational tasks based on characteristics of the input signal. Thanks to the mitochondrial regulations, the outcome signal possesses well-defined characteristics able to trigger specific programmes. At the same time, the adjustment of the parameters of the proposed mitochondrial PID controller (e.g. Ca^2+^ threshold) would change the cellular output. Translating this hypothesis to T cells, we expect multiple remodelling processes in the Ca^2+^ signalling network during activation, differentiation, apoptosis, etc. Multiple cytokines are crucial players in the regulation of the T cell states and we believe that their role is to provide cells with correct instructions, including how to remodel Ca^2+^ signalling patterns, in order to respond adequately to modifications of the environment. This view is confirmed by observations that T cell activation is associated with coordinated expression of RyR, IP3R, Orai1 together with MCU expression [131], while Ca^2+^ signalling in T cells is subjected to remodelling in terms of Ca^2+^ sources depending on the stage of activation [71].

The present model focuses on the roles of Ca^2+^ fluxes as key mediators of mitochondrial control of T cell activation. It does not provide a complete description of the molecular pathways responsible for T cell activation, which involve many other important factors such as nitric oxide, mitochondrial mass, mTOR signalling, etc. The model could be applied to other cell types owing to similarity in the fundamental mechanisms and principles of signalling regulation among different cell types. As an example, decoding of Ca^2+^ oscillations by cardiomyocytes and hypertrophic response are executed by the Ca^2+^-sensitive NFAT/CaN system, which can act as an integrator [164] in the same way as in T cells [94]. Further, recent observations indicate that an increase in Ca^2+^ concentration results in primary salivary gland epithelial cells acquiring an acinar-like phenotype. This process is associated with the remodelling of the Ca^2+^ signalling machinery: upregulation of Orai1, STIM1, STIM2 and NFAT1, accompanied by an elevation in SOCE [165]. According to our hypothesis, this remodelling might be considered necessary to fine-tune the Ca^2+^ response and the parameters of output of the PID controller that result in the realization of specific programmes. Finally, these conclusions might have not only fundamental interest, but also therapeutic implications, because they would allow us to predict which spots in cellular Ca^2+^ signalling have the most crucial effect in different pathological states.
